# THE MANIFESTATION OF MULTI-LEVEL STIGMA IN THE LIVED EXPERIENCES OF TRANSGENDER OLDER ADULTS

**DOI:** 10.1093/geroni/igz038.2722

**Published:** 2019-11-08

**Authors:** Vanessa Fabbre, Eleni Gaveras

**Affiliations:** Washington University in St. Louis, St. Louis, Missouri, United States

## Abstract

Transgender and gender nonconforming (TGNC) older adults experience disparities in mental health outcomes when compared to non-TGNC sexual minority older adults. Stigmatizing experiences are thought to influence these outcomes, but little is known about this process. Recent conceptualizations of stigma draw attention to multiple levels – individual, interpersonal, and structural – experienced by TGNC people of all ages. To explore how multi-level stigma manifests in the lives of TGNC older adults, we conducted a two-phase qualitative content analysis of in-depth biographical interviews with 88 TGNC adults aged 50 and older, from across the United States. Data were obtained from the photography and interview project To Survive on This Shore. Our interpretive analyses suggest that TGNC older adults’ development and well-being are impacted by multiple levels of stigma, which are dynamic and unpredictable, resulting in constant awareness of a changing social environment. Individual level stigma is experienced as ongoing vigilance about aspects of oneself that break gender norms, which is also marked by self-imposed social isolation and fears about accessing older adult services. At the interpersonal level, TGNC older adults navigate unpredictable interpersonal relationships, which manifest as fluctuating levels of love, acceptance, strain, and exclusion. Structural stigma manifests in the awareness of stigmatizing policies and systems but also in the conscious action of TGNC older adults to resist these structures. TGNC older adults promote supportive structural responses to stigma to both improve conditions for younger generations while also reducing experiences of individual and interpersonal stigma for themselves.

